# A74 A RARE CASE OF ANTIDEPRESSANT-INDUCED LYMPHOCYTIC COLITIS IN A TEENAGER

**DOI:** 10.1093/jcag/gwad061.074

**Published:** 2024-02-14

**Authors:** A Binaqail, V D Morinville, V Nguyen

**Affiliations:** McGill University Health Centre, Montreal, QC, Canada; McGill University Health Centre, Montreal, QC, Canada; McGill University Health Centre, Montreal, QC, Canada

## Abstract

**Background:**

The etiopathogenesis of microscopic colitis may reflect an immunological reaction in genetically predisposed individuals exposed to an external stimulus causing gut microbiota disruption. Numerous factors such as smoking, autoimmune diseases, and medications have been linked to this condition in adults. Steroids may be required as therapy. Pediatric cases of microscopic colitis have only rarely been reported.

**Aims:**

Case report.

**Methods:**

Retrospective single chart review and literature review of microscopic colitis in children.

**Results:**

A 17-year-old female newly diagnosed with major depression disorder following intentional acetaminophen intoxication was started on sertraline, a selective serotonin reuptake inhibitor (SSRI). Within 2 months from initiation, she began to have non bloody watery diarrhea and had a 5 kg weight loss over a 1-month period . Her laboratory workup only revealed a hypochromic, microcytic anemia. Due to severity of symptoms and reluctance of treating team to discontinue sertraline, she underwent an esophagogastroduodenoscopy and ileo-colonoscopy, both showing grossly normal mucosa. Histological assessment of the colon demonstrated increased intraepithelial lymphocytes, up to 50-60 lymphocytes/100 enterocytes (normal ≤ 20 lymphocytes/100 enterocytes), with associated reactive superficial epithelial changes including goblet cell and mucin depletion, compatible with lymphocytic colitis (Figure1). Sertraline was weaned off, and gastrointestinal symptoms resolved completely. A repeat sigmoidoscopy 5 months later confirmed complete normalization of the histology.

**Conclusions:**

Microscopic Colitis encompasses two disorders, lymphocytic colitis and collagenous colitis, in which the endoscopic appearance is unremarkable, but histological changes are diagnostic. The clinical manifestations of both are similar and typically include chronic non bloody watery diarrhea, weight loss and abdominal pain. Certain medications have been implicated in causing lymphocytic colitis, including SSRIs. SSRIs historically have not been frequently prescribed in pediatrics, but their use has increased in recent years. The complete resolution of symptoms and histology, without the need for any medical therapy, strongly supports that this lymphocytic colitis was caused by sertraline. The association of lymphocytic colitis with sertraline is not well-known among pediatric practitioners: neither the pediatrician nor psychiatrist in our case were aware of such a possibility. This case highlights that medication adverse events, even if rare, should always be considered when chronology is supportive, and at times, simple discontinuation of the drug is sufficient to completely resolve the issue.

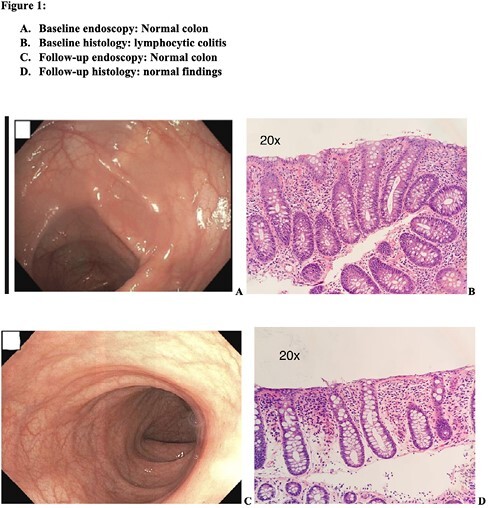

**Funding Agencies:**

None

